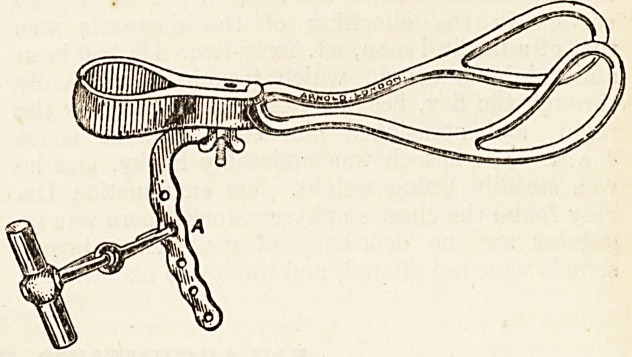# A Modification of Neville's Forceps

**Published:** 1910-02-19

**Authors:** G. C. Garratt


### A MODIFICATION OF NEVILLE'S FORCEPS.

### WITH PRACTICAL HINTS AS TO THEIR USE, AND A NOTE ON THE TREATMENT OF OCCIPITO-POSTERIOR PRESENTATIONS.

By G. C. GARRATT, M.D.

Some years ago while in Dublin I had the privilege of making the acquaintance of the late Dr. Neville, and, having, at the Rotunda Hospital, seen his forceps in regular use, I purchased a pair. Subsequently, however, after failing to deliver a large child in a case of slight pelvic contraction with this instrument, I nevertheless succeeded with a pair of Dr. Milne Murray's pattern. I therefore designed the forceps here figured in order to combine with the more accurate axis traction of the latter, the advantage peculiar to the former. My traction rod A is attached, precisely as by Dr. Neville, after application of the blades, a single screw serving both to fix it and to lock them. To insure axis traction, follow the movement of the curved rod A, and pull, as shown, always along the line of its extended radius. For normal pelves the hook is attached to the middle hole, for abnormal the other holes are available. Taking a hint from Dr. Porter Mathew, whom I had met when he was designing his forceps, I have slightly shortened the blades and also the handles, which are not meant for traction except at the final stage, the tractor being intended to be always used at other times. I have had these forceps now in use for four years and a half, and can recommend them as neat, portable (fitting into a 13-in. steriliser), and quite efficient both for the high and low operation. They are made by Messrs. Arnold and Sons, West Smithfield.

Having decided to use forceps in a suitable case, one has to choose the best position for the patient. I most strongly recommend the dorsal cross-bed position with the legs secured by simple webbing straps hooked to the rail at head and foot of the bed. Straps suitable for this purpose, yet small enough to be carried in the pocket, are figured in Messrs. Arnold's catalogue, and I have found them of the greatest service. When the patient is placed thus, with the buttocks well over the edge of the bed, mackintosh or brown paper underneath, and a pail below, cleanliness is insured, even if the rectum has not been completely emptied, the bed is kept dry even if a large douche is required, and the operator is most favourably placed for making an accurate diagnosis, for applying the forceps aseptically, and for dealing with any complication, *e.g.* hæmorrhage, ruptured perinæum, etc., that may arise. Not only so, but an assistant, always undesirable if uncertain in nerve or cleanliness, or if space is limited, is dispensed with, for no force that is justifiable will materially shift the patient, while, last but by no means least, her respiratory movements are unhampered, and are in full view of the operator. In fact, the lateral position has nothing to commend it, except custom, æsthetic considerations, and more convenient reception of the baby. As regards anæsthesia, if the lateral position be used, this will, in order to insure asepsis, be required to the full surgical degree, while if the dorsal position as described be chosen, a tolerant patient of the labouring class will often allow forceps to be applied, and be thereby delivered, without anæsthetic, with no more pain than in ordinary labour, but with considerable satisfaction at the improved progress made. In such a case traction should as far as possible be made only during the pains, which often become more strong and efficient as the head descends. Probably the use of scopolamine and morphia will enlarge the scope of this procedure; indeed, recently a primipara, who in the first stage of labour had been noisy and almost uncontrollable, and had then received gr. 1/100 and gr. ¼ respectively, was finally, to her great relief, delivered by the forceps without further anæsthesia and with little complaint. The child was of average size, and the perinæum was preserved. Where anæsthetic is necessary, as it will probably always be among the more sensitive classes, the patient, having been got under its influence by any method preferred, may readily be kept so; if an anæsthetist be not to hand, by the operator himself, using a Junker's inhaler with a face-piece, and extra long rubber tube on each side of the bottle to allow the latter to be hung on the bed rail, and the bellows to be worked on the ground with one foot. In this way chloroform, patient, and the effect of one on the other are under full control. and in full view of the responsible person, who nevertheless has both hands entirely disengaged. All the assistance he needs is someone well out of his way on the other side of the bed, to hold the face-piece in position. Lacks he even this help, perhaps a soft catheter in the nose might replace the face-piece. Since cases of persistent occipito-posterior presentation form a considerable fraction of those requiring assistance, and also, as I have been told, are not very rarely sent into hospital with a view to perforation after forceps have failed, a note on their diagnosis and treatment may not be out of place. Let us suppose that the second stage of labour is so protracted as to call for interference, fat and resistant abdominal walls obscure palpation, and moulding and caput succedaneum disguise sutures. Anæsthesia being induced if necessary, pass the whole hand into the vagina and feel for an ear; it will point to the occiput. Gently push up the head till this can be grasped; it can then also be rotated into its corresponding anterior position. According to Herman, this is possible even after long traction with forceps, but it is better to do it first. Next pass two fingers a little higher and rotate the most accessible shoulder to follow the head, which will then, and then only, remain in the corrected position; and finally apply the forceps at leisure. The above supplementary manœuvre which I happened to learn while at Queen Charlotte's Hospital from the resident medical officer, though no doubt it is often practised elsewhere, I have found easier, more certain, and more expeditious than rotation of the shoulder by external manipulation. The method often advocated of applying forceps to the head in the uncorrected position, and leaving the turns to nature, seems to me more tedious, more risky to mother and child, and less certain of success. It may lead to rupture of the perinæum into the rectum, or to the necessity for perforation where it might otherwise have been avoided.

## Figures and Tables

**Figure f1:**